# Exploring the impact of the Index of Multiple Deprivation on percutaneous coronary intervention outcomes: Insights from the British Cardiovascular Intervention Society database

**DOI:** 10.21542/gcsp.2025.4

**Published:** 2025-02-28

**Authors:** Sushant Saluja, Bernard D. Keavney, Mohammed Alawami, Magdi El-Omar, Freidoon Keshavarzi, Maaham Saleem, Scott Garg, Simon G. Anderson

**Affiliations:** 1 Division of Cardiovascular Sciences, Faculty of Biology, Medicine and Health, The University of Manchester, UK; 2 Division of Medicine and Manchester Academic Health Science Centre, Manchester University NHS Foundation Trust Manchester, Manchester, UK; 3 Department of Cardiology, Royal Blackburn Hospital, Blackburn, UK; 4 Caribbean Institute for Health Research, The University of the West Indies, Mona, Jamaica

## Abstract

**Background:** The effect of socioeconomic status on percutaneous coronary intervention (PCI) outcomes in populations with universal healthcare is poorly understood. Previous studies have primarily focused on ST-segment elevation myocardial infarction (STEMI) patients.

**Methods:** We analysed PCI outcomes from the British Cardiovascular Intervention Society database (2007–2014), categorised by deprivation quintiles. The primary endpoint was 30-day all-cause mortality, with hazard ratios calculated using Cox regression, adjusting for hospital clustering.

**Results:** Among 437,024 eligible patients, with 1.78 million person-years of follow-up, 39.9% underwent PCI for stable coronary artery disease (CAD), 38.4% for non-STEMI, and 21.6% for STEMI. During a median follow-up of 3.5 years, 52,258 patients (11.9%) died. Crude mortality rates increased with greater deprivation (from 26.7 per 1,000 person-years in the least deprived to 28.5 per 1,000 in the most deprived; *p* for trend <0.0001). Increased mortality rates with worsening IMD were observed only in patients treated for non-STEMI. Adjusted for various covariates, including age, sex and PCI indication, 30-day mortality rates were 14% higher (HR: 1.14; 95% CI:1.06 to 1.24; *p* <0.0001) in the most deprived patients compared to the least deprived. Similar patterns were observed for 1-year (HR:1.09; 95% CI:1.04 to 1.14) and 5-year mortality (HR:1.10; 95% CI:1.06 to 1.16).

**Conclusion:** Socioeconomic deprivation independently increases mortality risk after non-STEMI, but doesn’t affect outcomes for stable CAD or STEMI in universal healthcare settings. Targeted strategies are needed to address this disparity.

## Introduction

Socioeconomic disadvantage is often linked with disparities in health care quality and outcomes across various medical conditions^1,2^. Numerous studies have established a connection between low socioeconomic status (SES) and increased cardiovascular morbidity and mortality. Several factors may contribute to this disparity, including a higher prevalence of cardiovascular risk factors, unequal access to cardiac procedures such as angiography and percutaneous coronary intervention (PCI), and lower adherence to medical therapies among socioeconomically disadvantaged patients^3,4^.

The influence of socioeconomic status on outcomes from percutaneous coronary intervention in populations with universal healthcare access is not well-documented^5,6^. Previous research has yielded inconsistent results and has often relied on arbitrary measures of social deprivation, such as income or zip codes. Many studies have used only one or two indicators of SES, like household income or highest educational attainment^7,8^. However, health is influenced by a multitude of social and economic factors, suggesting that a composite measure might be more accurate. Moreover, many studies examining quality of care and outcomes based on SES have been conducted in countries without universal healthcare.

In the UK, where healthcare is universally accessible and free at the point of delivery, the Index of Multiple Deprivation (IMD) is used to measure relative deprivation. The IMD considers seven domains: income, employment, health and disability, education and training, housing and services, living environment, and crime^9^. This study aims to assess the relationship between socioeconomic status and outcomes following PCI within the UK’s universal healthcare system using this standardized marker of deprivation. By addressing inconsistencies in the literature and the global emphasis on reducing health disparities, we aim to determine the association between SES, baseline risk profile and clinical outcomes, in patients undergoing PCI for STEMI, NSTEMI, and stable angina, based on data from a large British registry.

## Methods

### Study design and population

This study is a retrospective analysis using data from the British Cardiovascular Intervention Society (BCIS) on percutaneous coronary interventions performed in the UK between 2007 and 2014. The dataset encompasses 113 variables, which include indications for PCI, detailed procedural information, and patient outcomes up to the point of hospital discharge. Data entry is performed exclusively by registered caregivers and clerks, with all information being encrypted and securely transmitted to centralised servers.

The BCIS database is subject to stringent validation processes to ensure data accuracy and completeness. Data collection adheres to established standardised protocols, with trained clinicians and data managers tasked with entering information at each participating institution^10^.

The data entry system is equipped with automated range checks, internal consistency algorithms, and error-flagging mechanisms to detect and reduce inconsistencies. In addition, periodic audits are carried out by the National Institute for Cardiovascular Outcomes Research (NICOR) to validate essential clinical variables against source medical records.

Previous analyses have established the reliability of BCIS data in accurately capturing procedural attributes, patient demographics, and long-term outcomes, supported by external validation studies that reaffirm its robustness within the realm of cardiovascular research^10^. Annual mortality tracking links each patient’s National Health Service number with the BCIS database and the Office of National Statistics (ONS), which records all UK deaths^10^.

Detailed data capture, handling, and validation procedures have been previously published^11^. Patients were stratified by quintile of the Index of Multiple Deprivation (IMD), from least deprived to most deprived. The patient population was followed from the procedure date until death or the study censor date (December 31, 2014).

### Outcome measures

The primary endpoint was all-cause mortality at 30 days. Secondary outcomes included mortality at 1 year and 5 years. Data was collected from recorded entries in the BCIS database.

### Statistical methods

To estimate the multivariable hazard ratios (HRs) for mortality within 30 days, 1 year, and 5 years, we employed Cox regression models with shared frailty or cluster models, focusing on the naïve cohort. Hazard ratios were calculated for each quintile relative to the least deprived quintile. We collected data on patient demographics and procedural characteristics. Continuous variables were evaluated as mean ± standard deviation (SD), with percentages referring to the cohort where data was available. Student’s *t*-test was used for continuous variables, and chi-squared testing assessed the significance of categorical variables. Multiple imputations were used to address missing values and reduce bias from a complete case-only analysis.

To address the missing data issue and mitigate potential bias inherent in a complete case-only analysis, we utilised multiple imputations by chained equations (MICE). This methodology is predicated on assuming data are missing at random (MAR). Imputation of missing values was performed using predictive mean matching for continuous variables and logistic regression for binary variables, thereby ensuring that the imputed values remained plausible within the structural confines of the dataset. A total of 20 imputed datasets were created to enhance the stability of the estimation process, with results subsequently pooled following Rubin’s rules to generate the final estimates. The imputation model encompassed all covariates pertinent to the primary analysis, including patient demographics, procedural characteristics, and clinical outcomes, thereby preserving the interrelationships among the variables. Rigorous diagnostic checks were conducted, including a comparison of the distributions of observed and imputed values and an assessment of convergence, to validate the efficacy of the imputation process.

Variables that were potential predictors of mortality were included in the analysis, such as age, gender, weight, diabetes, hypertension, smoking, cerebrovascular disease (transient ischaemic attack or stroke), peripheral vascular disease (PVD), renal impairment (defined as serum creatinine > 200 μmol/L or on dialysis), warfarin use, cardiogenic shock, hypercholesterolemia, previous myocardial infarction (AMI), previous PCI, previous coronary artery bypass grafting (CABG), Q wave on ECG, valvular heart disease, left ventricular ejection fraction, angina status pre-surgery, and procedural details [including indication for PCI, pre- and post- procedure TIMI flow, route of access, oral antiplatelet agent used (i.e., prasugrel, ticagrelor or clopidogrel), anti-thrombotic agent used (heparin, heparin and bivalirudin, heparin and GP IIb/IIIa inhibitor), presence of shock pre-procedure, use of inotropes, arterial complications, major bleeding, ventilated pre-procedure, left main stem (LMS) culprit lesion, use of thrombectomy, and use intra-aortic balloon pump (IABP)]. Variables found to be significant (*p* <0.05) on univariate analysis were consequently included in multivariable Cox regression.

All statistical analyses were conducted using STATA software version 18^12^. All *p*-values were two-tailed, with a significance threshold set at *p* <0.05.

## Results

Between 2007 and 2014, there were a total of 670,170 PCI procedures performed in the UK. After excluding individuals without IMD data, missing mortality data, multiple admissions (131,966), non-English participants (100,716), and participants at the extremes of ages (<17 or >105) (464), there were 437,024 participants with data available for analysis. Among these eligible patients with 1.78 million person-years of observation, 40% had PCI for stable angina, 37.7% for non-STEMI, and 21.6% for STEMI.

The distribution according to deprivation from least (Q1) to most (Q5) deprived was: Q1 (18.0%); Q2 (20.0%); Q3 (20.0%); Q4 (19.7%); Q5 (22.3%).

During the study period, the mortality rate at a median 3.5-year follow-up (with an interquartile range of 1.8−5.5 years) was 11.9% (*n* = 52,258). After adjusting for covariates in the multivariable Cox regression analysis—including radial access, anti-thrombotic use, hypercholesterolemia, hypertension, age, sex, previous PCI, smoking history, arterial complications, diabetes, Q-waves on ECG, peripheral vascular disease, valvular heart disease, history of renal disease, and use of circulatory support—it was found that 30-day mortality rates were higher among patients from the most deprived areas compared to those from the least deprived areas. However, the increase was only significant for patients with a non-ST elevation myocardial infarction (NSTEMI). These trends were consistent at 1-year follow-up, and 5-year of follow up.

**Figure 1.**
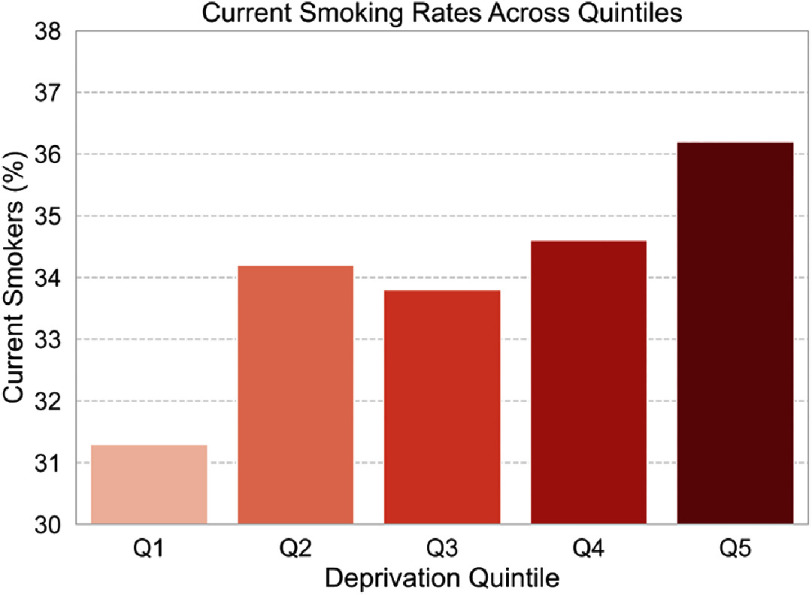
Percentage of current smokers across quintiles of socioeconomic deprivation (Q1 being the least deprived, Q5 being the most deprived). The chart illustrates a clear trend of increasing smoking prevalence with higher levels of deprivation.

Significant variations were observed in the distribution of age, with patients in Q5 being younger (60.5 years) compared to Q1 (64.3 years) (*p*¡0.001). Gender distribution showed minimal differences across quintiles. Patients in Q5 exhibited higher rates of prior myocardial infarction (56.7% vs. 54.8%, *p* < 0.001) and hypertension (68.8% vs. 69.9%, *p* < 0.001) compared to Q1. Diabetes mellitus was more prevalent in Q5 for oral medication/dietary control (29.5% vs. 27.9%, *p* < 0.001) and insulin therapy (8.7% vs. 9.4%, *p* < 0.001). Chronic renal failure requiring dialysis was also more common in Q5 (5.3% vs. 3.1%, *p* < 0.001). Conversely, a history of coronary artery bypass grafting (CABG) was less frequent in Q5 compared to Q1 (9.5% vs. 11.2%, *p* = 0.032), while end-stage renal disease (ESRD) was more prevalent in Q5 (6.5% vs. 4.7%, *p* < 0.001). There were no significant differences in the distribution of left ventricular ejection fraction (LVEF) across quintiles. Similarly, the proportions of current smokers were higher in Q5 than Q1 (Figure 1). Target vessels for percutaneous coronary intervention (PCI) showed no significant differences between Q1 and Q5. Essentially, those in the most deprived quintile (Q5) are characterized by a younger age at presentation, higher prevalence of prior myocardial infarction, hypertension, diabetes, and chronic renal failure. Conversely, they exhibit lower rates of prior CABG but higher rates of ESRD compared to patients in the least deprived quintile (Q1). See Table 1 for more information on this.

**Table 1:** Clinical characteristics of ACS patients stratified by quintiles of deprivation. Values are mean ± SD.

**Variable**	**Quintile 1 (Q1)**	**Quintile 2 (Q2)**	**Quintile 3 (Q3)**	**Quintile 4 (Q4)**	**Quintile 5 (Q5)**	***P*-value (trend)**
Total number of patients	78,670 (18.0%)	87,405 (20.0%)	87,405 (20.0%)	86,069 (19.7%)	97,475 (22.3%)	<0.001
STEMI	16,028 (20.4%)	18,506 (21.2%)	18,889 (21.6%)	18,879 (21.9%)	22,095 (22.7%)	<0.001
Stable Angina	31,465 (40.0%)	34,962 (40.0%)	34,962 (40.0%)	34,276 (39.8%)	39,144 (40.2%)	<0.001
NSTEMI	31,177 (39.6%)	33,937 (38.8%)	33,554 (38.4%)	32,914 (38.2%)	36,236 (37.2%)	<0.001
Age (years)	64.3 ± 11.2	63.1 ± 11.6	62.4 ± 10.9	61.8 ± 10.7	60.5 ± 10.4	<0.001
Sex (male)	55,820 (71.0%)	65,650 (75.1%)	65,230 (74.6%)	65,684 (76.3%)	73,883 (75.8%)	0.067
Previous MI	40,876 (52.0%)	47,403 (54.2%)	48,373 (55.3%)	47,053 (54.7%)	55,283 (56.7%)	0.041
Previous CABG	8,815 (11.2%)	9,715 (11.1%)	9,115 (10.4%)	9,613 (11.2%)	9,215 (9.5%)	0.032
Previous PCI	42,103 (53.5%)	50,821 (58.1%)	50,321 (57.6%)	50,921 (59.2%)	63,258 (64.9%)	0.043
Previous Stroke	8,172 (10.4%)	9,715 (11.1%)	9,315 (10.7%)	9,715 (11.3%)	10,952 (11.2%)	<0.001
Diabetes (oral medicine/dietary control)	21,935 (27.9%)	24,754 (28.3%)	24,354 (27.9%)	24,854 (28.9%)	28,722 (29.5%)	<0.001
Diabetes (insulin)	6,844 (8.7%)	7,666 (8.8%)	7,366 (8.4%)	7,666 (8.9%)	9,162 (9.4%)	<0.001
Chronic renal failure: dialysis	2,428 (3.1%)	2,622 (3.0%)	2,222 (2.5%)	2,822 (3.3%)	5,166 (5.3%)	<0.001
End stage renal disease	3,715 (4.7%)	4,370 (5.0%)	4,270 (4.9%)	4,570 (5.3%)	6,336 (6.5%)	<0.001
Known Hypercholesterolaemia	49,128 (62.4%)	52,243 (59.8%)	52,443 (60.0%)	52,143 (60.6%)	52,643 (54.0%)	0.335
Known Hypertension	54,125 (68.8%)	61,183 (70.0%)	61,183 (70.0%)	61,683 (71.7%)	68,135 (69.9%)	<0.001
Known PVD	11,144 (14.2%)	12,236 (14.0%)	12,436 (14.2%)	12,036 (14.0%)	12,236 (12.6%)	0.03
Left ventricular ejection fraction						
Good (LVEF >50%)	29,917 (40.1%)	36,502 (41.8%)	36,202 (41.4%)	36,602 (42.5%)	39,087 (40.1%)	0.342
Moderate (30–50%)	22,288 (30.1%)	26,422 (30.2%)	26,122 (29.9%)	26,522 (30.8%)	26,122 (26.8%)	0.435
Poor (<30%)	14,659 (20.0%)	17,681 (20.2%)	17,381 (19.9%)	17,781 (20.7%)	21,209 (21.8%)	0.261
Not measured	7,829 (10.6%)	8,440 (9.7%)	8,740 (10.0%)	8,640 (10.0%)	9,455 (9.7%)	0.243
Smoking status						
Current smoker	24,717 (31.3%)	29,918 (34.2%)	29,518 (33.8%)	29,818 (34.6%)	35,286 (36.2%)	0.025
Ever smoked (current + ex-smoker)	48,618 (61.8%)	52,643 (60.2%)	52,243 (59.7%)	52,643 (61.1%)	60,109 (61.7%)	0.221
Never	25,645 (34.2%)	34,962 (40.0%)	34,562 (39.5%)	34,962 (40.6%)	40,306 (41.3%)	<0.001
Valvular heart disease	4,358 (6.2%)	5,244 (6.0%)	5,344 (6.1%)	5,144 (6.0%)	5,725 (5.9%)	0.434
NYHA functional class >3	8,715 (11.1%)	9,615 (11.0%)	9,515 (10.9%)	9,715 (11.3%)	9,815 (10.8%)	0.11

**Notes.**

CABG coronary artery bypass graft CAD coronary artery disease LVEF Left Ventricular Ejection Fraction NSTEMI non-ST-segment elevation myocardial infarction NYHA New-York Heart Association PCI percutaneous coronary intervention STEMI ST-segment-elevation myocardial infarction

From a procedural perspective, patients from the most deprived quintile (Q5) underwent radial access procedures more frequently (10.6% vs. 8.5% in Q1, *p* < 0.0001) and presented with higher rates of cardiogenic shock before interventions (0.9% vs. 0.3% in Q1, *p* < 0.0001), indicating more severe disease presentations. Despite these challenges, Q5 patients exhibited higher rates of TIMI 3 flow both pre-procedure (4.4% vs. 3.9% in Q1, *p* < 0.0001) and post-procedure (26.8% vs. 8.1% in Q1, *p* < 0.0001), suggesting effective revascularisation outcomes. Moreover, they received more intensive antithrombotic therapies, including heparin (39.5% vs. 31.8% in Q1, *p* < 0.0001) and glycoprotein IIb/IIIa inhibitors (15.1% vs. 11.0% in Q1, *p* < 0.0001), as well as greater use of circulatory support such as intra-aortic balloon pumps (3.6% vs. 2.6% in Q1, *p* < 0.0001). Additionally, Q5 patients were more likely to have multi-vessel disease (6.0% vs. 4.5% in Q1, *p* = 0.03). See Table 2 for more information on this.

**Table 2:** Procedural and clinical characteristics of patients stratified by quintiles. Values are presented as number (percentage).

**Variable**	**Quintile 1 (Q1)**	**Quintile 2 (Q2)**	**Quintile 3 (Q3)**	**Quintile 4 (Q4)**	**Quintile 5 (Q5)**	***P*-value (trend)**
Total number of patients	78,670 (100%)	87,405 (100%)	87,405 (100%)	86,069 (100%)	97,475 (100%)	<0.0001
STEMI	12,589 (16.0%)	14,841 (17.0%)	15,350 (17.6%)	16,437 (19.1%)	19,803 (20.3%)	<0.0001
Stable Angina	24,375 (31.0%)	27,684 (31.7%)	28,500 (32.6%)	29,124 (33.8%)	31,612 (32.4%)	<0.0001
NSTEMI	30,177 (38.4%)	30,937 (35.4%)	31,054 (35.5%)	32,714 (38.0%)	34,036 (34.9%)	0.08
Q-wave on presenting ECG	5,100 (6.5%)	6,875 (7.9%)	8,000 (9.2%)	8,035 (9.3%)	8,988 (9.2%)	0.12
Arterial access						
Femoral	2,555 (3.2%)	2,640 (3.0%)	2,422 (2.8%)	2,576 (3.0%)	2,623 (2.7%)	0.07
Radial	6,706 (8.5%)	8,498 (9.7%)	9,060 (10.4%)	9,476 (11.0%)	10,348 (10.6%)	<0.0001
Pre-procedure TIMI flow						
0	3,119 (4.0%)	3,660 (4.2%)	4,100 (4.7%)	4,130 (4.8%)	4,860 (5.0%)	<0.0001
1	752 (1.0%)	1,044 (1.2%)	1,331 (1.5%)	1,410 (1.6%)	1,625 (1.7%)	<0.0001
2	1,395 (1.8%)	1,842 (2.1%)	2,022 (2.3%)	2,290 (2.7%)	2,775 (2.9%)	<0.0001
3	3,059 (3.9%)	3,558 (4.1%)	3,714 (4.2%)	3,950 (4.6%)	4,330 (4.4%)	<0.0001
Cardiogenic shock pre-procedure	262 (0.3%)	402 (0.5%)	496 (0.6%)	537 (0.6%)	838 (0.9%)	<0.0001
Vessel attempted						
LM	142 (0.18%)	202 (0.23%)	230 (0.26%)	237 (0.28%)	241 (0.25%)	0.11
LAD	3,479 (4.4%)	4,190 (4.8%)	4,640 (5.3%)	4,970 (5.8%)	5,580 (5.7%)	0.48
RCA	2,416 (3.1%)	3,400 (3.9%)	3,460 (4.0%)	3,780 (4.4%)	3,970 (4.1%)	0.23
LCx	1,925 (2.5%)	2,850 (3.3%)	2,915 (3.3%)	3,065 (3.6%)	3,760 (3.9%)	0.06
Grafts	405 (0.5%)	655 (0.8%)	748 (0.9%)	737 (0.9%)	763 (0.8%)	0.14
MVD	3,519 (4.5%)	4,275 (4.9%)	4,615 (5.3%)	4,935 (5.7%)	5,900 (6.0%)	0.03
Anti-thrombotic agent used						
Heparin only	25,051 (31.8%)	29,784 (34.1%)	32,495 (37.2%)	34,940 (40.6%)	38,530 (39.5%)	<0.0001
Heparin + GPIIb/IIIa	8,641 (11.0%)	11,678 (13.4%)	12,578 (14.4%)	13,365 (15.5%)	14,745 (15.1%)	<0.0001
Bivalirudin	3,107 (3.9%)	4,285 (4.9%)	4,448 (5.1%)	4,570 (5.3%)	5,485 (5.6%)	0.14
Warfarin	1,200 (1.5%)	1,780 (2.0%)	2,062 (2.4%)	2,222 (2.6%)	2,562 (2.6%)	0.12
Any circulatory support						
IABP used	2,031 (2.6%)	2,878 (3.3%)	3,056 (3.5%)	3,200 (3.7%)	3,535 (3.6%)	<0.0001
Ventilated	1,336 (1.7%)	1,901 (2.2%)	2,086 (2.4%)	2,250 (2.6%)	2,573 (2.6%)	0.018
Post-procedure TIMI flow						
0	892 (1.1%)	1,085 (1.2%)	1,171 (1.3%)	1,251 (1.5%)	1,472 (1.5%)	0.04
1	216 (0.3%)	293 (0.3%)	339 (0.4%)	393 (0.5%)	468 (0.5%)	0.03
2	1,020 (1.3%)	1,233 (1.4%)	1,317 (1.5%)	1,391 (1.6%)	1,536 (1.6%)	<0.0001
3	6,342 (8.1%)	7,555 (8.6%)	8,073 (9.2%)	8,475 (9.8%)	26,123 (26.8%)	<0.0001

**Notes.**

ACS acute coronary syndrome ECG electrocardiogram IABP intra-aortic balloon pump LAD left anterior descending artery LCx left circumflex artery LM left main artery MVD multivessel disease NSTEMI non-ST-segment elevation myocardial infarction PCI percutaneous coronary intervention RCA right coronary artery STEMI STsegment-elevation myocardial infarction TIMI Thrombolysis in Myocardial Infarction GPIIb/IIIa glycoprotein IIb/IIIa inhibitor

**Figure 2.**
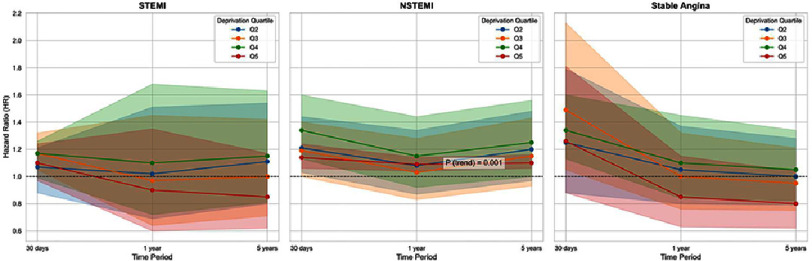
Mortality hazard ratios (HR) over time (30 days, 1 year, 5 years) stratified by deprivation quintiles (Q2-Q5) for STEMI, NSTEMI, and Stable Angina. The plots illustrate the relationship between socioeconomic deprivation and mortality risk across different cardiac conditions. Notably, NSTEMI demonstrates a statistically significant trend (*P* for trend = 0.001) of increasing mortality with higher deprivation, as indicated by the lines moving upwards with increasing quintile number. Shaded areas represent 95% confidence intervals.

**Figure 3.**
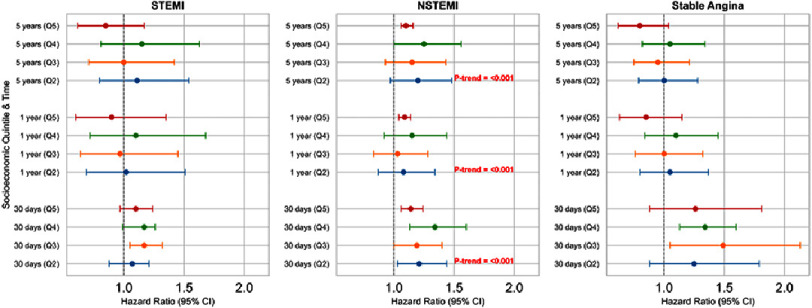
Visual representation of how socioeconomic deprivation affects mortality outcomes in patients who have suffered from various types of acute coronary syndromes (ACS). The forest plots within the figure display the hazard ratios for mortality at three key time points: 30 days, 1 year, and 5 years following the ACS event. These plots are further categorised by increasing levels of socioeconomic deprivation, ranging from quintile 2 (Q2) to quintile 5 (Q5), with Q5 representing the highest level of deprivation. Examining the individual ACS conditions reveals the impact of deprivation on mortality over time. In NSTEMI, a clear and concerning trend emerges. A statistically significant trend (*P* for trend <0.001) indicates that as socioeconomic deprivation increases, mortality risk rises across all time points –30 days, 1 year, and 5 years post-ACS. This visualisation emphasises the disproportionate burden faced by socioeconomically disadvantaged individuals, particularly those with NSTEMI. It underscores the critical need for healthcare interventions and policies that actively address these disparities to improve outcomes for the most vulnerable patients in our healthcare system.

Among 437,024 eligible patients, with 1.78 million person-years of follow-up, 39.9% underwent PCI for stable coronary artery disease (CAD), 38.4% for non-STEMI, and 21.6% for STEMI. During a median follow-up of 3.5 years, 52,258 patients (11.9%) died. Crude mortality rates increased with greater deprivation (from 26.7 per 1,000 person-years in the least deprived to 28.5 per 1,000 in the most deprived; > trend <0.0001). Increased mortality rates with worsening IMD were observed only in patients treated for non-STEMI. Adjusted for various covariates, including age, sex and PCI indication, 30-day mortality rates were 14% higher (HR: 1.14; 95% CI: 1.06 to 1.24; *p* < 0.0001) in the most deprived patients compared to the least deprived. Similar patterns were observed for 1-year (HR: 1.09; 95% CI: 1.04 to 1.14) and 5-year mortality (HR: 1.10; 95% CI: 1.06 to 1.16). Data is presented in Table 3 and visualised as mortality trends in Figure 2 and a forest plot in Figure 3.

**Table 3:** Hazard ratios (HRs) with 95% confidence intervals (CIs) and p-values for patients with different heart conditions. ST-segment elevation myocardial infarction [STEMI], non-ST-segment elevation myocardial infarction [NSTEMI], and stable angina), stratified by socioeconomic status (SES) quintiles (Q2-Q5), using Q1 as the reference (or base) group. Quintile 1 (Q1) represents the most advantaged SES group, while quintile 5 (Q5) represents the most disadvantaged. HRs are shown for three periods: 30 days, 1 year, and 5 years following the index PCI.

**Condition**	**Period**	**Metric**	**Q2**	**Q3**	**Q4**	**Q5**	***P*-value (trend)**
**STEMI**	30 days	HR (95% CI) *p*-value	1.07 (0.88–1.21) 0.220	1.17 (1.05–1.32) 0.006	1.17 (0.99–1.26) 0.070	1.10 (0.97–1.24) 0.110	*P* = 0.116
	1 year	HR (95% CI) *p*-value	1.02 (0.69–1.51) 0.918	0.97 (0.64–1.45) 0.866	1.10 (0.72–1.68) 0.669	0.90 (0.60–1.35) 0.620	*P* = 0.142
	5 years	HR (95% CI) *p*-value	1.11 (0.80–1.54) 0.542	1.00 (0.71–1.42) 0.980	1.15 (0.81–1.63) 0.439	0.85 (0.62–1.17) 0.319	*P* = 0.125
**NSTEMI**	30 days	HR (95% CI) *p*-value	1.21 (1.03–1.44) 0.020	1.19 (1.00–1.40) 0.040	1.34 (1.13–1.60) <0.001	1.14 (1.06–1.24) <0.001	*P* < 0.001
	1 year	HR (95% CI) *p*-value	1.08 (0.87–1.34) 0.488	1.03 (0.83–1.28) 0.796	1.15 (0.92–1.44) 0.225	1.09 (1.04–1.14) 0.030	*P* < 0.001
	5 years	HR (95% CI) *p*-value	1.20 (0.97–1.48) 0.085	1.15 (0.93–1.43) 0.205	1.25 (1.00–1.56) 0.041	1.10 (1.06–1.16) 0.025	*P*<0.001
**Stable Angina**	30 days	HR (95% CI) *p*-value	1.25 (0.88–1.79) 0.220	1.49 (1.05–2.13) 0.030	1.34 (1.13–1.60) 0.010	1.26 (0.88–1.81) 0.200	*P* = 0.117
	1 year	HR (95% CI) *p*-value	1.05 (0.80–1.37) 0.723	1.00 (0.76–1.32) 0.989	1.10 (0.84–1.45) 0.495	0.85 (0.63–1.15) 0.315	*P* = 0.115
	5 years	HR (95% CI) *p*-value	1.00 (0.79–1.28) 0.983	0.95 (0.75–1.21) 0.702	1.05 (0.82–1.34) 0.694	0.80 (0.62–1.04) 0.089	*P* = 0.143

## Discussion

This research provides important insights into the link between socioeconomic deprivation and cardiovascular outcomes, particularly in the context of universal healthcare. Our analysis of a large real-world dataset highlights that deprivation is a significant independent indicator of mortality in patients with non-ST-elevation myocardial infarction. This finding is consistent with previous research, which suggests that patients undergoing percutaneous coronary intervention with a higher social deprivation index (SDI) are more likely to have existing comorbidities and face an increased risk of all-cause mortality^2,7^.

The findings indicate that the differential outcomes observed between the most and least deprived quintiles are relatively modest, quantified at merely 14%. This observation prompts a pertinent inquiry: what accounts for the minimality of this discrepancy? Moreover, the absolute difference translates to an increment of merely 2 additional fatalities per 1000 patients over the span of one year, suggesting that the overall outcomes within our healthcare system are not as acute as one may presume when considering the socioeconomic disparities.

This observation indicates that our healthcare system may be effectively alleviating some adverse consequences associated with lower socioeconomic status in the context of STEMI and stable angina. Nevertheless, it is evident that substantial efforts remain necessary. Consequently, it is imperative to address cardiovascular risk factors and bolster primary prevention strategies within communities of low socioeconomic status to enhance health outcomes.

Furthermore, the concept that lower socioeconomic status groups can experience adverse health outcomes even in the presence of universal healthcare warrants investigation, particularly concerning the elevated mortality rates associated with NSTEMI in these populations, compared to the more favourable outcomes observed in patients with STEMI and stable coronary artery disease. In cases of NSTEMI, the phenomenon of delayed presentation, which is prevalent among lower SES groups, is particularly detrimental. Unlike STEMI, characterized by acute symptoms that prompt immediate medical intervention such as percutaneous coronary intervention, NSTEMI symptoms are often less pronounced. This can lead patients to postpone seeking necessary medical care^13^. Furthermore, individuals with limited access to healthcare services or lower health literacy levels may experience significant myocardial damage prior to receiving treatment due to this delay. The management of NSTEMI typically involves pharmacological therapy and selective PCI, rendering early intervention less uniform and more susceptible to delays, especially among disadvantaged populations^14^.

The observed disparity in outcomes for non-ST-elevation myocardial infarction across different socioeconomic strata can be attributed to several interrelated mechanisms. A pivotal factor is the delayed healthcare-seeking behaviour prevalent in lower socioeconomic status populations, which stems from diminished symptom awareness, competing socio-economic priorities, and barriers to navigating the healthcare system^15^.

In contrast to ST-elevation myocardial infarction, which typically presents with sudden and severe symptoms that necessitate prompt medical intervention, the symptoms of NSTEMI are often more subtle and ambiguous. This subtlety results in extended symptom-to-door times, consequently leading to increased myocardial damage before appropriate intervention can be initiated^16^.

Furthermore, lower health literacy and reduced healthcare engagement within socioeconomically disadvantaged communities exacerbate the likelihood of missed or delayed diagnoses. Research indicates that patients from lower SES backgrounds are less likely to participate in regular primary care consultations, resulting in a deficit of early risk stratification and suitable secondary prevention measures. This deficiency in early intervention disproportionately impacts NSTEMI patients, as their treatment pathway is significantly contingent upon timely pharmacological management and scheduled percutaneous coronary intervention, rather than emergent reperfusion strategies^17,18^.

Conversely, patients presenting with STEMI typically receive expedited, aggressive treatment because of the urgent need for reperfusion, which minimises the adverse effects associated with delayed presentation, even within marginalised groups^14^. Therefore, outcomes for STEMI patients are generally more favourable.

In the case of stable CAD or stable angina, while lower SES may negatively influence access to routine healthcare, the slower progression of the disease means that patients are not confronted with the same immediate risks posed by NSTEMI. Consequently, socioeconomic factors are less pronounced in exacerbating outcomes in stable disease states. In contrast, the interaction of delayed care and more intricate management protocols in NSTEMI significantly contributes to the observed disparities in mortality rates.Top of Form

The relationship between social determinants of health and increased comorbidities among cardiovascular patients is well-documented^19,20^. The interaction between social and biological systems, where socioeconomic disparities contribute to poor health outcomes, offers a plausible explanation. Limited access to nutritious food, transportation, and safe environments for physical activity are commonly cited factors^4,7^. Interestingly, our study did not find differences in hyperlipidaemia rates between patients with higher and lower SDI, suggesting that other underlying mechanisms might be at play, such as reverse causality where poor health exacerbates socioeconomic disparities, leading to financial instability and residence in more deprived neighbourhoods.

Prior studies have emphasized socioeconomic status (SES) as a factor in prognosis following PCI^6,7^. Our focus on the influence of community-level SES, revealed that patients in the highest SDI quintile were more likely to have had prior PCI but less likely to have had prior coronary artery bypass grafting (CABG). In our study cohort, individuals from lower socioeconomic status backgrounds did exhibit significantly higher incidences of smoking, hypertension, and prior coronary heart disease (CHD). These results align with those observed in previous cohorts. Numerous cohort studies have demonstrated that disparities in risk factors account for over half of the increased cardiovascular event risk observed in lower SES groups^21–23^.

While SES provides a broad measure of community health, it may not capture individual patient nuances. Nevertheless, it can be useful for identifying patients who require further screening for social risks. Mitigation strategies, such as deploying dedicated social workers and patient navigators, could help target resources to at-risk individuals. Our study’s strength lies in its large, diverse sample from various community settings, although limitations exist. The study was limited to a single healthcare system, potentially limiting its generalizability to other systems, especially those with different demographics or resources. Additionally, our reliance on medical records constrained our ability to incorporate external factors not captured by SDI.

It has been suggested that poorer outcomes for lower socioeconomic status groups may persist even in universal healthcare systems. This could be due to a lower awareness of myocardial infarction symptoms and delays in seeking medical help^24^. In our study, patients who arrived late might have died before receiving percutaneous coronary intervention or might not have undergone PCI at all. Nationally, the most common reason for not providing reperfusion therapy is late presentation, usually more than 12 h after symptoms start^25^. Therefore, the surviving patients from lower SES groups presenting with STEMI might represent a more selective and less risky group, potentially leading to survivorship bias.

To address these disparities, adopting a multifaceted approach that incorporates community-driven interventions, transformative health system reforms, and the integration of digital health technologies is imperative. One viable strategy involves the incorporation of cardiovascular risk screening programs within primary care settings and community health centres located in socioeconomically disadvantaged areas. These initiatives should prioritise the early detection of non-ST elevation myocardial infarction by utilising high-sensitivity troponin assays and establishing structured pathways for chest pain assessment^15^. Moreover, amplifying the involvement of pharmacists and community healthcare workers in these regions can significantly enhance medication adherence and patient education, particularly concerning dual antiplatelet therapy and lipid-lowering treatment regimens.

On a more extensive scale, implementing mobile health (mHealth) interventions—such as automated SMS reminders aimed at improving medication adherence, digital symptom checkers, and telemedicine consultations—can mitigate access barriers faced by individuals contending with transportation or time limitations. Additionally, policies that offer financial incentives to hospitals for implementing targeted outreach programs and for incorporating screenings of social determinants of health into standard cardiovascular evaluations could further promote equity in the management of NSTEMI. By systematically addressing both clinical and non-clinical impediments to care, these strategies are poised to significantly diminish socioeconomic status-based disparities in cardiovascular health outcomes^26^.

## Limitations of the study

Since this study is retrospective, there is a potential for unidentified confounding factors. Excluding non-English participants (*n* = 100,716) may introduce selection bias and constrain the generalisability of our findings. It is crucial to recognise that non-English-speaking individuals may encounter distinct healthcare challenges, including language barriers that adversely affect healthcare access, symptom recognition, and treatment adherence. Consequently, the disparities identified in our study may not comprehensively reflect the experiences of specific ethnic or migrant populations. Future research should investigate these disparities using alternative datasets that more effectively account for linguistic and cultural variances in healthcare utilisation^27^.

Another limitation is that SES was assessed using the Index of Multiple Deprivation (IMD), an area-based measure. Area-based measures can result in the “ecologic fallacy”, where individual SES is misclassified based on the neighbourhood’s SES. However, this can be offset by avoiding the ”individualistic fallacy”, which incorrectly assumes an individual’s health is not influenced by their neighbourhood^28^. Furthermore, SES can change over time, a factor we could not account for, though the contributions of adult and early life factors to social inequalities in coronary heart disease remain uncertain^24,29^.

Our findings showed no significant differences in clinical outcomes post-PCI for ST-elevation myocardial infarction across different SES groups. This is despite lower SES patients having more baseline cardiovascular risk factors. An observed slight increase in stroke rates among lower SES groups may reflect a higher baseline prevalence of cerebrovascular disease rather than SES alone. In the context of universal healthcare, disparities in access to timely reperfusion therapy can influence outcomes. Other studies conducted in countries with universal healthcare systems, including Switzerland, Italy, and Denmark, have yielded similar findings. One study indicated that patients with lower socioeconomic status had higher mortality rates, but this difference was not statistically significant after adjusting for age. The other two studies found no significant differences in mortality related to SES. Our study aligns with these findings, demonstrating no disparities in outcomes following STEMI based on socioeconomic status within a universal healthcare system^30–32^.

In the United Kingdom, access to and choice of drug-eluting stents (DES) are not influenced by socioeconomic status, unlike in many other countries^33,34^. Historical funding restrictions and insurance status may have previously affected DES usage in places with insurance-based healthcare systems. However, these disparities are expected to diminish over time as costs decrease and funding policies evolve. In contrast, the National Health Service (NHS) ensures that all patients receive the best possible care, regardless of their SES, by providing equitable access to DES and other treatments. Compliance with medical therapy is another critical factor, with lower SES groups usually showing less adherence to guideline-recommended therapies, potentially due to higher rates of contraindications^35–37^.

## Conclusion

Our study suggests that in a universal healthcare context, SES is not an independent predictor of adverse outcomes following PCI for STEMI but might influence outcomes in the context of an NSTEMI. Therefore, reducing the burden of cardiovascular risk factors and improving primary prevention in low SES communities remains crucial. Addressing these disparities through targeted public health initiatives could significantly improve long-term cardiovascular health outcomes.

## Funding

None.

## Competing interests

The authors declare that they have no competing interest.

## Author statements

Data Curation: Sushant Saluja, and Simon G. Anderson

Formal Analysis: Sushant Saluja, Maaham Saleem, and Simon G. Anderson

Writing - Original Draft: Sushant Saluja, Bernard D. Keavney, Maaham Saleem, Mohammed Alawami, Simon G. Anderson, Freidoon Keshavarzi, and Magdi El-Omar

Supervision: Simon G. Anderson, Bernard D. Keavney, Mohammed Alawami, Scot Garg, Magdi El-Omar

Writing - Review & Editing: All authors

Project Administration: All authors

Validation: All authors

Conceptualisation: All authors

## Acknowledgement

S.S. is supported by the 4Ward North Wellcome Trust Clinical Research Training Fellowship (Grant Reference 203914/*Z*/16/*Z*). S.G.A. was supported by an NIHR.

## References

[1] Kivimäki M, Batty GD, Pentti J (2020). Association between socioeconomic status and the development of mental and physical health conditions in adulthood: A multi-cohort study. The Lancet Public Health.

[2] Schultz WM, Kelli HM, Lisko JC (2018). Socioeconomic status and cardiovascular outcomes. Circulation.

[3] Rosengren A, Smyth A, Rangarajan S (2019). Socioeconomic status and risk of cardiovascular disease in 20 low-income, middle-income, and high-income countries: The Prospective Urban Rural Epidemiologic (PURE) study. The Lancet Global Health.

[4] Davari M, Maracy MR, Khorasani E (2019). Socioeconomic status, cardiac risk factors, and cardiovascular disease: A novel approach to determination of this association. ARYA Atheroscler.

[5] Biswas S, Andrianopoulos N, Duffy SJ (2019). Impact of socioeconomic status on clinical outcomes in patients with ST-segment–elevation myocardial infarction. Circulation: Cardiovascular Quality and Outcomes.

[6] Denvir MA, Lee AJ, Rysdale J (2006). Influence of socioeconomic status on clinical outcomes and quality of life after percutaneous coronary intervention. J Epidemiol Community Health.

[7] Torabi AJ, Lohe EVonder, Kovacs RJ, Frick KA, Kreutz RP (2023). Measures of social deprivation and outcomes after percutaneous coronary intervention. Catheterization and Cardiovascular Interventions.

[8] Anand VV, Zhe ELC, Chin YH (2023). Socioeconomic deprivation and prognostic outcomes in acute coronary syndrome: A meta-analysis using multidimensional socioeconomic status indices. International Journal of Cardiology.

[9] Abel GA, Barclay ME, Payne RA (2016). Adjusted indices of multiple deprivation to enable comparisons within and between constituent countries of the UK including an illustration using mortality rates. BMJ Open.

[10] Ludman P (2019). British Cardiovascular Intervention Society database: Insights into interventional cardiology in the United Kingdom. Heart.

[11] Banning AP, Baumbach A, Blackman D (2015). Percutaneous coronary intervention in the UK: Recommendations for good practice 2015. Heart.

[12] New in Stata 18 — Stata. https://www.stata.com/new-in-stata/.

[13] Fu R, Song CX, Dou KF (2019). Differences in symptoms and pre-hospital delay among acute myocardial infarction patients according to ST-segment elevation on electrocardiogram: An analysis of China Acute Myocardial Infarction (CAMI) registry. Chinese Medical Journal.

[14] 2023 ESC Guidelines for the management of acute coronary syndromes. https://www.escardio.org/Guidelines/Clinical-Practice-Guidelines/Acute-Coronary-Syndromes-ACS-Guidelines.

[15] Nadarajah R, Farooq M, Raveendra K (2023). Inequalities in care delivery and outcomes for myocardial infarction, heart failure, atrial fibrillation, and aortic stenosis in the United Kingdom. The Lancet Regional Health –Europe.

[16] Differences in symptom presentation and hospital mortality according to type of acute myocardial infarction - PubMed. https://pubmed.ncbi.nlm.nih.gov/22520522/.

[17] Health literacy, social determinants of health, and disease prevention and control - PMC. https://pmc.ncbi.nlm.nih.gov/articles/PMC7889072/.

[18] Shahid R, Shoker M, Chu LM, Frehlick R, Ward H, Pahwa P (2022). Impact of low health literacy on patients’ health outcomes: A multicenter cohort study. BMC Health Services Research.

[19] Xia M, An J, Safford MM (2024). Cardiovascular risk associated with social determinants of health at individual and area levels. JAMA Network Open.

[20] Teshale AB, Htun HL, Owen A (2023). The role of social determinants of health in cardiovascular diseases: An umbrella review. Journal of the American Heart Association.

[21] Alter DA, Naylor CD, Austin P, Tu JV (1999). Effects of socioeconomic status on access to invasive cardiac procedures and on mortality after acute myocardial infarction. N Engl J Med.

[22] Osler M, Gerdes LU, Davidsen M (2000). Socioeconomic status and trends in risk factors for cardiovascular diseases in the Danish MONICA population, 1982–1992. J Epidemiol Community Health.

[23] Kaplan GA, Keil JE (1993). Socioeconomic factors and cardiovascular disease: A review of the literature. Circulation.

[24] Steele L, Palmer J, Lloyd A, Fotheringham J, Iqbal J, Grech ED (2019). Impact of socioeconomic status on survival following ST-elevation myocardial infarction in a universal healthcare system. Int J Cardiol.

[25] Herrett E, Smeeth L, Walker L, Weston C (2010). on behalf of the MINAP Academic Group. The Myocardial Ischaemia National Audit Project (MINAP). Heart.

[26] Taking action on the social determinants of health in clinical practice: A framework for health professionals - PMC. https://pmc.ncbi.nlm.nih.gov/articles/PMC5135524/.

[27] Whitaker KL, Krystallidou D, Williams ED (2022). Addressing language as a barrier to healthcare access and quality. Br J Gen Pract.

[28] Diez-Roux AV (1998). Bringing context back into epidemiology: Variables and fallacies in multilevel analysis. Am J Public Health.

[29] Emberson JR, Whincup PH, Morris RW, Walker M (2004). Social class differences in coronary heart disease in middle-aged British men: Implications for prevention. Int J Epidemiol.

[30] Jakobsen L, Niemann T, Thorsgaard N (2012). Dimensions of socioeconomic status and clinical outcome after primary percutaneous coronary intervention. Circulation: Cardiovascular Interventions.

[31] Fournier S, Muller O, Ludman AJ, Lauriers N, Eeckhout E (2013). Influence of socioeconomic factors on delays, management and outcome amongst patients with acute myocardial infarction undergoing primary percutaneous coronary intervention. Swiss Med Wkly.

[32] Gnavi R, Rusciani R, Dalmasso M (2014). Gender, socioeconomic position, revascularization procedures and mortality in patients presenting with STEMI and NSTEMI in the era of primary PCI. Differences or inequities?. Int J Cardiol.

[33] Gaglia MA, Torguson R, Xue Z (2010). Insurance type influences the use of drug-eluting stents. JACC: Cardiovascular Interventions.

[34] Vicuna R, House J, Spertus J, Kao J (2006). Abstract 3992: Effect of insurance status on use of drug eluting stents versus bare metal stents in patients undergoing elective PCI. Circulation.

[35] Wilder ME, Kulie P, Jensen C (2021). The impact of social determinants of health on medication adherence: A systematic review and meta-analysis. J Gen Intern Med.

[36] Ekenberg M, Qvarnström M, Sundström A, Martinell M, Wettermark B (2024). Socioeconomic factors associated with poor medication adherence in patients with type 2 diabetes. Eur J Clin Pharmacol.

[37] Lee H, Park JH, Floyd JS, Park S, Kim HC (2019). Combined effect of income and medication adherence on mortality in newly treated hypertension: nationwide study of 16 million person-years. Journal of the American Heart Association.

